# When to Consider Immune Checkpoint Inhibitors in Oncogene-Driven Non-Small Cell Lung Cancer?

**DOI:** 10.1007/s11864-019-0652-3

**Published:** 2019-06-06

**Authors:** Laurent Mhanna, Nicolas Guibert, Julie Milia, Julien Mazieres

**Affiliations:** 1 Pulmonology Department, Toulouse University Hospital, Université Paul Sabatier, Toulouse, France; 2 Thoracic Oncology Department, Hopital LARREY, CHU Toulouse, Chemin de Pouvourville, 31059 Toulouse, France

**Keywords:** Immunotherapy, Oncogenic addiction, Targeted therapy, *EGFR*, *ALK*, Non-small cell lung cancer

## Abstract

Targeted therapies and more recently immune checkpoint inhibitors (ICI) have transformed the treatment landscape of advanced NSCLC. Clinical trials investigating immune checkpoint inhibitors (ICI) have usually excluded patients with oncogenic drivers, so that the outcome of these agents in this population is poorly known. In patients with oncogenic addiction, targeted therapy remains clearly the best option, and the place of immunotherapy in this population has not been clearly defined yet.

Based on available data, we suggest that (i) immunotherapy single agent should be proposed only after exhaustion of more validated treatments, (ii) combinations of immunotherapy with targeted therapies are of interest provided that we can manage toxicity and find the best sequence, (iii) a combination of immunotherapy with chemotherapy may be appealing in patients pretreated with targeted agents. The best way to opt in for the best strategy will depend upon the identification of adequate biomarkers. New basic and clinical research is awaited in this field.

## Introduction

Lung cancer is the main cause of cancer-related mortality worldwide. Its management underwent significant transformation over the past 10–15 years leading to two new therapeutic strategies: targeted therapies and immunotherapy. Genotype-directed treatments targeting oncogenic addictions (*EGFR*, *BRAF*, and *HER2* mutations, or *ALK* and *ROS1* rearrangements) demonstrated high response rates and prolonged PFS.

Immunotherapy has been also recently developed in non-small cell lung cancer (NSCLC). The positive results of clinical trials assessing PD-1 and PD-L1 inhibitors in both metastatic and locally advanced stages revolutionized the treatment landscape of NSCLC [1–3]. In all the studies considering these agents however, only a minority of patients (15–20%) derived a durable benefit and a new pattern of hyperprogressive patients has been identified, suggesting an urgent need for new biomarkers of both response and resistance.

The place taken by ICI in patients with oncogenic driver is actually debated, since they have been usually excluded from phase 3 immunotherapy trials. However, despite a usual dramatic and durable activity of targeted therapies, resistance inexorably develops [1]. Treatment options upon exhaustion of targeted therapies are limited, underscoring the need to explore the potential interest of ICI in these populations.

Another challenge is to further understand the biological drivers of inflammation and immune escape in NSCLC with oncogenic addiction and to identify subgroups deriving benefits.

We aim herein to review the main data available regarding the outcome of patients harboring an oncogenic addiction and treated with ICI either alone, in combination with chemotherapy or concomitantly to targeted therapy.

## What is the immunogenicity of lung cancer with oncogenic addiction?

The evolution of tumors bearing a molecular alteration usually depends on a single dominant mechanism following the principle of oncogenic addiction, which has been described as the dependence of tumor cells upon the specific activity of an activated oncogene [4]. A single mutation or translocation is supposed to confer a survival advantage to the respective cells and it is usually isolated, explaining the low tumor mutation burden observed in these tumors [5], leading to less inflamed tumor microenvironments with death of tumor-infiltrating CD8^+^ lymphocytes, explaining the low response rate to PD-1 inhibitors observed amongst *EGFR*- and *ALK*-driven NSCLC [6••].

**Fig. 1:**
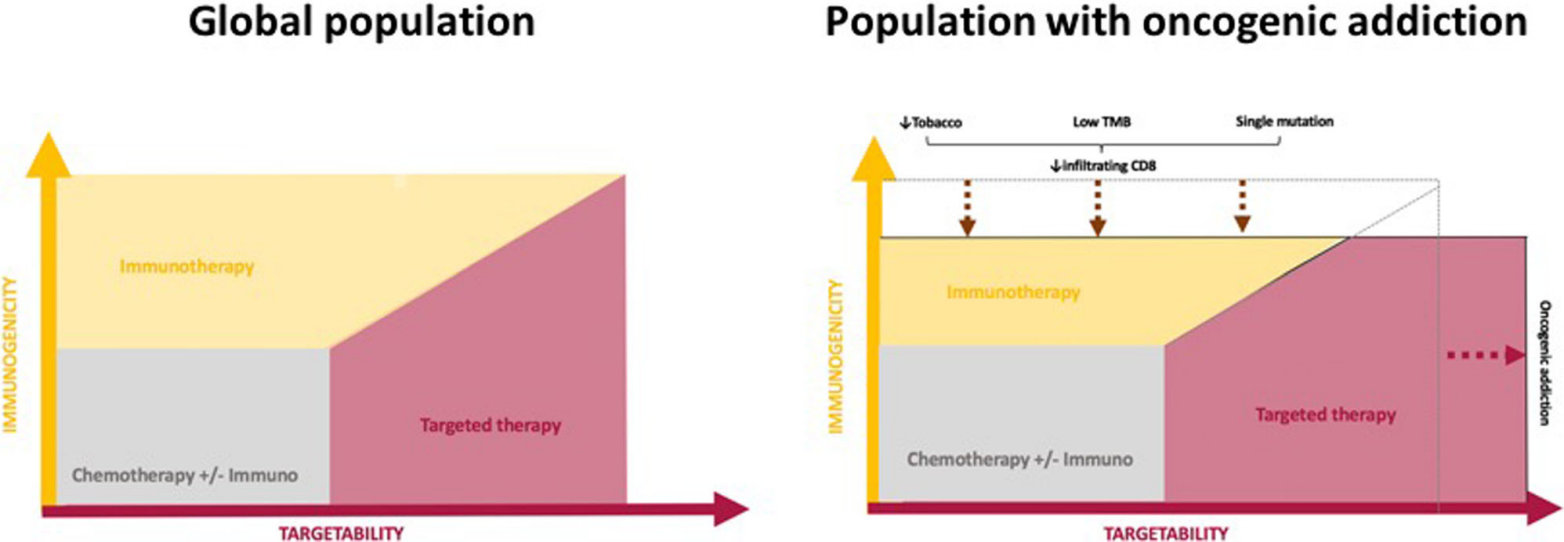
Immunogenicity and sensitivity to targeted agents in situation of oncogenic addiction.

**Fig. 2:**
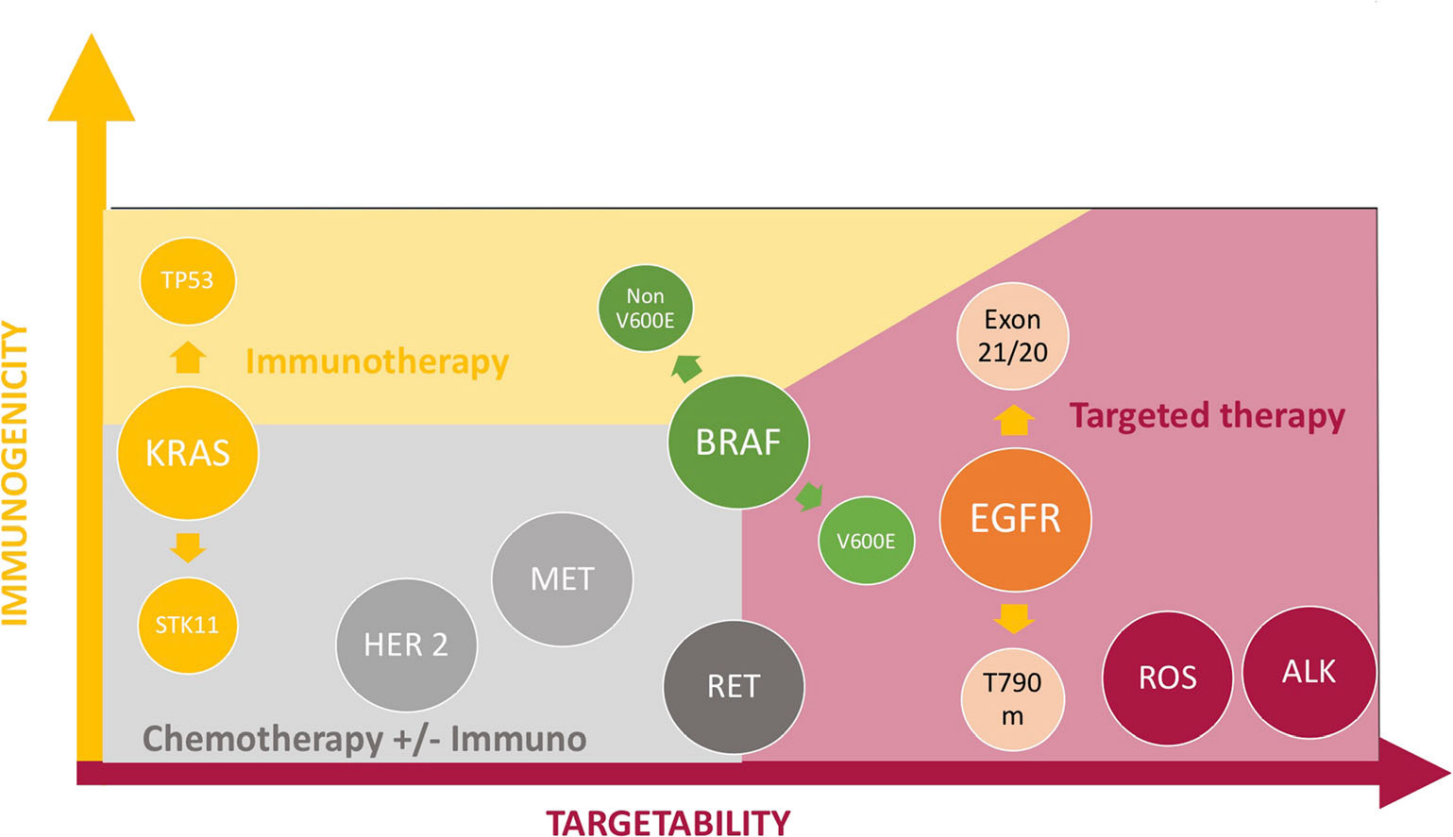
Likelihood of sensitivity to ICI and/or genotype-directed agents in each oncogenic addiction setting.

*EGFR*, *BRAF*^*v600E*^ mutations, *ALK*, *ROS*, or *RET* rearrangements but also *MET* ^exon 14^ mutations are usually found in non-smokers, in contrast with *KRAS* and BRAF non V600E which are more likely found in smokers [7]. It is clearly established that tobacco exposure is associated with higher tumor mutation burden (TMB) and correlates with higher responsiveness to ICI, while non-smokers will less likely respond to these agents due to excluded, non-inflamed microenvironments [6••, 8–10]. Figure 1 and Figure 2 summarize the sensitivity to ICI and targeted therapy regarding each oncogenic addiction.

## Anti-tumor activity of ICI as monotherapy according to each type of mutation

### KRAS

*KRAS* mutations are the most frequent molecular alterations encountered in advanced NSCLC and are correlated with both poor prognosis and poor outcomes under chemotherapy [11–13]. Moreover, all studies involving therapies targeting KRAS failed to demonstrate any clinical benefit up to now [14, 15] due notably to the complexity of its downstream signaling pathways.

ICI however clearly represent an attractive alternative in *KRAS*-mutated patients, since correlations between *KRAS* mutations and sustained responses to checkpoint inhibitors have systematically been reported in trials [1, 8] . In a recent work, Miao et al. reported that clonal driver alterations in *KRAS* were associated with complete or partial response to ICI [16]. This relationship is likely due to a strong epidemiologic association with tobacco that generates a high mutation burden [17, 18].

In our retrospective IMMUNOTARGET cohort, we reported an interesting 3.2 months of median PFS in the overall *KRAS* population but failed to detect a significant impact of the different *KRAS* mutations subtypes on PFS or OS [19••]. PD-L1 expression in this population was associated with better outcomes*.*

More interestingly, some molecular alterations have been shown to have a direct impact, independently on their epidemiologic relationship with tobacco, on the tumor microenvironment. In particularly, *STK11* mutations, inactivated in 20 to 30% of adenocarcinomas, and often associated with *KRAS* mutations, are associated with non-inflamed tumor microenvironment in a murine model. *STK11* inactivation leads to the an increased infiltrate of protumoral neutrophils, a decrease of tumor-infiltrating cells and reduced PD1 and PD-L1 expressions in the tumor microenvironment, both in a *KRAS*-driven mouse model of lung adenocarcinoma and in cell lines [15]. Furthermore, tumors mutated for both *KRAS* and *TP53* (KP) have a higher mutation load than tumors co-mutated for *KRAS*/*STK11* (KL), and KP tumors are characterized by a stronger inflammatory response [20, 21]. In agreement with these preclinical signals, recent data showed that *TP53* and *STK11* may also accurately stratify patients between responders (57% ORR for *KRAS-P53* tumors) and non-responders (0% ORR in *KRAS-STK11* tumors) in the Checkmate 057 trial [22•].

### EGFR

Regarding *EGFR*, contradictory results have been reported in preclinical studies [23] However, clinical studies have systematically reported disappointing results. Gainor et al. identified a very low response rate (3.6%) in patients harboring EGFR mutations treated by immunotherapy and showed an association with non-inflamed tumor microenvironment [6••]. A recent meta-analysis involving three randomized trials of immunotherapy in pretreated patients (CheckMate 057, Keynote 010, and POPLAR) reported that ICI did not improve overall survival compared with docetaxel in *EGFR*-mutated patients (HR 1.11; 95% CI 0.80–1.53, *p* = 0.54, interaction *p* = 0.005) [24•]. In a recent phase II study of pembrolizumab in PD-L1 positive, TKI-naïve *EGFR*-mutated patients presented no response even in tumors with high (≥ 50%) PD-L1 expression highlighting that frontline ICI is inappropriate in EGFR patients [25]. A phase 2, open-label, single-arm trial (ATLANTIC) studied the efficacy of durvalumab, a PD-L1 inhibitor, in pretreated NSCLC including 111 patients with *EGFR* or *ALK* alteration [26•]. Patients with *EGFR* or *ALK* alteration had previously received standard treatment with tyrosine kinase inhibitors. Amongst the 111 oncogene addicted patients, 77 presented PD-L1 expression in at least 25% of tumor cells. The aimed response rate was 12.2% in oncogene addicted patients with PD-L1 expression > 25% of tumor cells, while in patients with < 25% PD-L1 expression, the objective response rate was only 3.6%. Progression-free survival was 1.9 months and was not influenced by PD-L1 expression.

Several explanations to these poor outcomes have been identified, such as an association of EGFR mutation with low TMB and a lack of T cell infiltration [6••, 27] while PD-L1 expression is variable [26•, 27•, 28•].

Our IMMUNOTARGET cohort support these findings, with however some differences between sub groups. PFS was 1.4 months for the T790M attached subgroup; 1.8 months for exon 19 deletions; 2.5 for exon 21 mutations, and 2.8 for other mutations (*p* < 0.0001) [21]. In contrast, Negrao et al presented appealing data with exon 20 *EGFR*-mutated tumors compared with classic *EGFR*-mutations and demonstrated higher OR and disease control rates at 6 and 12 months respectively 13% and 4% versus 6% and 0% for classic *EGFR* group) but also higher PFS (2.9 vs 1.9 months HR 0.45, *p* = 0.002) and OS (HR 0.2, *p* < 0.001), compared with common *EGFR* mutations [29]. Further studies are requested to confirm these differences in outcomes between *EGFR* mutations subtypes and to adjust these outcomes to tobacco use (more frequent amongst exon 20 mutated patients) and ideally TMB.

### ALK, ROS, and other translocations

Few studies included *ALK* patients and all reported very poor outcome under ICI. However, the number of patients was usually very low, precluding definitive conclusions [1, 6••, 8, 26•].

Other translocations, such as *RET* or *ROS1*, have been less studied. Poor results were found in the IMMUNOTARGET cohort amongst the concerned population. *ALK*, *ROS1*, and *RET* were analyzed together in a “rearrangement” subgroup, showing a 4.9% ORR (2/30) [21].

ICI outcomes in *RET*-rearranged patients have been studied elsewhere. In another retrospective cohort, it was found that *RET* rearrangements were associated with low TMB and poor response to immunotherapy compared with unselected patients [30•]. Altogether, even if data for *ALK /ROS /RET* translocations are preliminary and concern a low number of patients, we do not recommend ICI as single agents in patients with *ALK/ROS1/RET* rearranged NSCLC. Further studies are needed in order to see if these patients will benefit from combination therapies.

### BRAF mutation

The efficacy of immune checkpoint inhibitors in *BRAF*-mutated NSCLC has also been scarcely studied. Indeed, none of the large clinical trials evaluating anti-PD1/anti-PD-L1 agents in advanced NSCLC patients reported results in this specific subgroup. In a retrospective study of 39 *BRAF* mutant patients, Dudnik et al. showed interesting results of immunotherapy with median PFS of 3.7 and 4.1 months and response rates of 25 and 33% in patients with V600E and no V600E mutations, respectively. Of note, no significant difference was found between V600E and non-V600E patients in this study [31•].

The IMMUNOTARGET database, however, allowed us to collect data from 35 *BRAF*-mutated patients who received immunotherapy, of which 17 (48.5%) were V600E. In this cohort, *BRAF* mutations were associated with slightly better results compared with *EGFR* mutations: ORR was 24% and median PFS of 3.1 months. In *BRAF* patients, PFS was influenced by smoking, with smokers having better PFS than non-smokers. It therefore seems that immunotherapy should be considered in BRAF-mutated patients, especially if they are smokers. Moreover, non-V600E mutations tended to be associated with better response rates and PFS than V600E mutations, likely due its epidemiologic association with tobacco use compared with V600E patients [19••].

### MET alteration

In a recent study investigating ICI outcomes in *MET* exon 14-altered lung cancers, ORR was 17% (4/24) and median PFS was 1.9 months (95% CI 1.7–2.7). The authors concluded that responses to ICI may be achieved, but overall clinical efficacy remains modest, suggesting that targeted therapies and chemotherapy must be also favored in this population [32]. The IMMUNOTARGET *MET* exon 14 cohort proved better outcomes with a median PFS of 4.7 months [20]. This result is supported by another recent limited series (*n* = 8) from Dudnik et al. in which the median PFS was 4.0 months (95% CI, 2.4–NR). However, the limited number of patients analyzed does not allow us to draw definitive conclusions.

## How to obtain better results?

Except for KRAS patients, response rates to immunotherapy used as a single agent in all oncogene addiction situations were globally shown to be impaired compared with wild-type patients [19••, 24•, 33, 34]. We forecast two main strategies to optimize the integration of immunotherapy in this population: the first one is to identify more reliable biomarkers. The second one is to use them combined with other drugs.

### Biomarkers

#### PD-L1

Currently, PD-L1 expression remains the most reliable predictive biomarker to guide PD-1 inhibitors treatment. A recent meta-analysis has clearly demonstrated its interest (OR 2.51), even using a cut-off of 1% (OR 2.17) [35]. The value of this biomarker has been clearly proved in most of second-line trials but also in the frontline setting, with pembrolizumab showing its superiority over platinum doublet chemotherapy in terms of progression-free but also overall survival in patients whose tumor strongly expressed PD-L1 (≥ 50%), establishing it as a new standard of care [3]. In the IMMUNOTARGET study, PD-L1 expression was significantly associated not only with better outcomes in both *EGFR* and *KRAS* subgroups but also in the whole population [22•].

PD-L1 expression is however challenging to interpret in cases of oncogenic addictions. PD-L1 expression may be induced by the oncogenic signaling pathways, but it is not necessarily associated with a strong immune cells infiltration [36, 37] . Furthermore, PD-L1 is a dynamic biomarker, which expression could be induced by radiation therapy, chemotherapies [38, 39], or targeted therapies. Data from archived tissue, often used to guide treatment decisions, are not necessarily representative of the tumor microenvironment at the time of ICI initiation. In conclusion, PD-L1 appears to be less reliable in this context and should be combined with other biomarkers more representative of the tumor microenvironment in order to better select accurate candidates.

#### Tumor mutation burden

Beyond PDL1 expression, simple clinical factors have also been explored as predictors of response to immune checkpoint inhibitors. In particular, a lack of tobacco exposure has been associated with lower responsiveness to PD1 blockade [6••, 40]. One explanation for these findings is that lung cancer in never or minimal smokers is generally associated with a low TMB [10], another predictive biomarker of response to immune checkpoint inhibitors, independent of PDL1 expression [41] . Low TMB results in a lack of immunogenic neo-antigens, and thus non-inflamed (“excluded”) microenvironment. This is the most likely explanation to the most favorable outcomes observed in the *KRAS, BRAF* non-V600E, and even *MET* exon14 patients, alterations that are more frequently observed in smokers. This is consistent with other studies reporting low TMB in *EGFR, ALK, ROS1* [42], or *RET*-driven lung cancers [30•], but higher in *KRAS* or *BRAF* [42].

TMB is however, like PD-L1, a dynamic biomarker that may be modified by many anti-cancer treatments. In the KEYNOTE-189 and PACIFIC trials, for example, never-smokers derived benefits from immunotherapy [2, 43], likely because chemotherapy and radiation therapy dramatically change the tumor microenvironment, leading to immunogenic cell death with neoantigens release and local and systemic T cells expansions. It is likely that these changes, combined with DNA alterations (and therefore increased TMB), explain the benefit observed in never smokers. TMB analysis can thus be a reliable marker in this population. However, whole-exome sequencing (WES) or the use of broad NGS panels, mandatory for TMB calculation, might be challenging to be translated into routine clinical practice due to the cost, the lack of a standardized panel and cut-off, and the limited availability of tissue [41]. There is thus a need to move beyond TMB and identify specific genetic determinants of response to PD-1 inhibitors, especially since not all point mutations will result in the genesis of highly immunogenic peptides [44].

The best predictive marker of response in this population combined with TMB may in fact be the tumor-infiltrating lymphocytes (TILs), which defines the notion of “hot” inflammatory tumor and “cold,” excluded, tumor.

### Combination therapies

To increase the probability to obtain sustained disease control in patients with oncogene addiction using ICI, combination therapies may be a promising approach [45]. Activating the patient’s immune system during the time of tumor reduction and remission may be the best way to ensure that responses are converted into long-term and durable benefits. Targeted therapy could enhance anti-tumor immune responses by releasing neoantigens and synergistically improve of ICI anti-tumor activity [46]. From a clinical point of view, the combination of targeted agents with immunotherapies is of interest, considering that immunotherapy may transform the important tumor responses achieved with small molecule inhibitors to durable and long-lasting remissions. For example, in melanoma it has been proved that BRAF inhibition could have favorable effects in the tumor microenvironment and it becomes more immunogenic [47]. Several clinical trials investigating combination strategies are currently ongoing, especially for EGFR and ALK. First-line therapy combination of nivolumab and erlotinib showed excellent response rates, even in EGFR–TKI pretreated patients, but with a grade 3–4 incidence of adverse-events of 24% [48]. A phase 1 trial showed an ORR of 77.8% with gefitinib + durvalumab (*n* = 10) vs 80% with gefitinib alone followed by the combination of the two drugs (*n* = 10) [49]. Osimertinib and durvalumab also showed encouraging outcomes in TKI-naïve or resistant EGFR-mutated patients, but this combination seems to strongly potentialize the risk of interstitial pneumonia.

There is also a clear rational for a synergistic effect of ICI, chemotherapy, and anti-VEGF, even in cases of oncogenic addiction. Indeed, cytotoxic chemotherapy exposes the immune system to a high tumor antigen load and the normalization of tumor microvascularisation by anti-VEGF induces an increase in T lymphocyte infiltration [50]. Such a combination strategy has recently been analyzed in the ImPOWER 150 study (platine-based chemotherapy, bevacizumab, and atezolizumab) and showed significant efficacy in patients with oncogene addictions after failure of all available generations of TKI. The odds ratio for overall survival between the two groups was 0.54 (0.29; 1.03).

## Conclusion

**Fig. 3:**
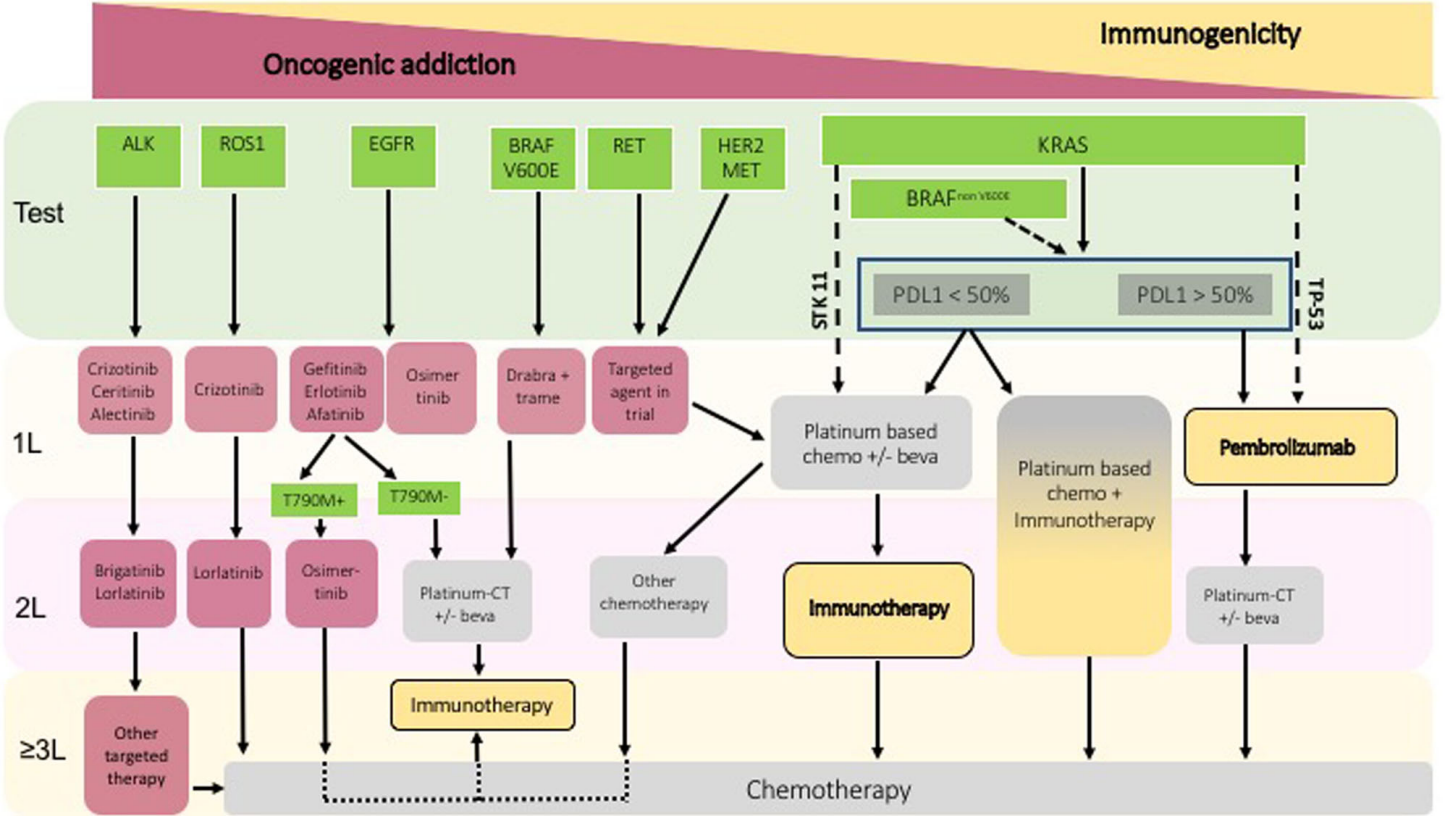
Proposed algorithm of integration of immunotherapy in the management of patients with oncogenic addiction.

Lung cancer is becoming a more diverse disease with regard to management including a wide range of targets and treatment options. In patients with oncogenic addiction, targeted therapy is clearly the most suitable option. The potential interest of immunotherapy to improve patients’ outcomes and particularly long-term survival has not been defined yet. Based on published work, we suggest that (i) immunotherapy single agent should be proposed only after exhaustion of much validated treatments (an algorithm according to each driver is proposed in Fig. 3), (ii) a combination of immunotherapy with targeted therapy is of interest provided that we can manage toxicity and find the best sequence, (iii) a combination of immunotherapy with chemotherapy may be appealing in patients pretreated with targeted agents. The best way to opt in for the best strategy will depend upon the identification of adequate biomarkers. New basic and clinical research is awaited in this field.
